# Translating evidence-based interventions for implementation: Experiences from Project HEAL in African American churches

**DOI:** 10.1186/1748-5908-9-66

**Published:** 2014-05-31

**Authors:** Cheryl L Holt, Erin K Tagai, Mary Ann Scheirer, Sherie Lou Z Santos, Janice Bowie, Muhiuddin Haider, Jimmie L Slade, Min Qi Wang, Tony Whitehead

**Affiliations:** 1 Department of Behavioral and Community Health, University of Maryland, School of Public Health, 2369 School of Public Health (Building 255), College Park, MD 20742, USA; 2 Scheirer Consulting, Princeton, USA; 3 Department of Health, Behavior & Society, Johns Hopkins Bloomberg School of Public Health, Baltimore, USA; 4 Maryland Institute for Applied Environmental Health, University of Maryland, School of Public Health, College Park, USA; 5 Community Ministry of Prince George’s County, Upper Marlboro, USA; 6 Department of Anthropology, University of Maryland, College Park, USA

**Keywords:** Implementation, Dissemination, Evidence-based, Faith-based, African American, Cancer, Health communication, Technology

## Abstract

**Background:**

Community-based approaches have been increasing in the effort to raise awareness and early detection for cancer and other chronic disease. However, many times, such interventions are tested in randomized trials, become evidence-based, and then fail to reach further use in the community. Project HEAL (Health through Early Awareness and Learning) is an implementation trial that aims to compare two strategies of implementing evidence-based cancer communication interventions in African American faith-based organizations.

**Method:**

This article describes the community-engaged process of transforming three evidence-based cancer communication interventions into a coherent, branded strategy for training community health advisors with two delivery mechanisms. Peer community health advisors receive training through either a traditional classroom approach (with high technical assistance/support) or a web-based training portal (with low technical assistance/support).

**Results:**

We describe the process, outline the intervention components, report on the pilot test, and conclude with lessons learned from each of these phases. Though the pilot phase showed feasibility, it resulted in modifications to data collection protocols and team and community member roles and expectations.

**Conclusions:**

Project HEAL offers a promising strategy to implement evidence-based interventions in community settings through the use of technology. There could be wider implications for chronic disease prevention and control.

## Background

Cancer is a leading cause of death worldwide [1]. Community-based approaches have been increasing in the effort to raise awareness and screening [2,3]. Faith-based settings have been established as an effective community venue to reach underserved populations with health information [2-6]. However, many times, such interventions are tested in randomized trials, become evidence-based, and then fail to reach further implementation [7].

### Dissemination/implementation research

It is well-documented in cancer control and other areas, that a significant gap exists between research and practice [7]. It is insufficient to make evidence-based interventions available and assume that they will disseminate themselves into practice [8]. Previous research has reported on what makes for effective dissemination/implementation [9]. It was found that passive diffusion techniques such as mailing materials were in general not effective. However, active techniques that were more disseminational in nature such as train-the-trainer, media campaigns, and educating opinion leaders were more likely to be effective, particularly when used in combination. There has been a call for more research on community-based interventions [10]. Similarly, there was a general disconnect between intervention research and approaches to disseminate programs into the community, and an ‘urgent need for more research into dissemination of effective cancer control interventions’ [9]. Peer educators were viewed as a promising strategy that merited further study [9]. Glasgow and colleagues [7] concluded that efficacy trials are often so tightly controlled that the findings are usually not generalizable, and recommended that interventions be tested in real-world settings. Interventions that involve community stakeholders as true partners [8] and that are designed for broader implementation [10] are more likely to be disseminated/implemented, and intervention research should be guided by theory.

The church-based dietary project PRAISE! provides an excellent example of such an intervention, and was designed with institutionalization and sustainability in mind [11]. A comprehensive process evaluation was conducted based on several data collection instruments from multiple perspectives (*e.g*., Pastor, participant, health leader, county coordinator). Pastors also completed a survey to determine organizational capacity factors specific to the church. Pilot testing of the process evaluation plan was recommended as one of the lessons learned. This type of research is needed to discover the best ways to translate evidence into practice, including interventions in faith-based settings that use community-engaged research methods [12]. Dissemination/implementation research is fundamental in determining the mechanisms underlying successful implementation of interventions, particularly those serving culturally and ethnically diverse populations [8].

### Lay community health advisor interventions

Community health advisors (CHAs) play a significant role in health promotion in underserved communities. Lay persons have a unique ability to foster a trusting relationship between healthcare agencies and community members. CHAs have demonstrated their effectiveness in promoting health among groups lacking access to adequate care [13-16]. Ethnically, linguistically, socio-economically, and experientially indigenous to the communities in which they work, these trusted ‘insiders’ serve as cost-effective conduits of information, resources and services to medically underserved populations [13,17-20]. CHAs have been used to address a broad range of health issues [14,15], and a number of studies have illustrated the ability of CHAs to do effective prevention work, reduce cultural and linguistic barriers to care, help patients successfully navigate complex health systems, and improve the quality and cost-effectiveness of care [13].

Some studies using CHAs report on outcomes specific to the CHA. For example, a lay CHA program in breast cancer screening for Chinese-English bilingual individuals resulted in significant increases in CHAs’ knowledge and self-efficacy [21]. Another intervention reported outcomes of training lay Filipino CHAs to conduct small-group sessions to increase colorectal cancer screening [22]. The training resulted in significant pre-post increases in knowledge about colorectal cancer screening guidelines and self-efficacy.

Other CHA studies report on outcomes specific to those the CHA is intervening upon. In a lay CHA intervention for diffusing breast and cervical cancer screening information to Latinas through a series of 12 educational group sessions, knowledge about breast and cervical cancer and self-reported use of screening tests increased; however, mammography use did not increase significantly [23]. The Deep South Network used a CHA model to build a grassroots community infrastructure of ‘Community Health Advisors as Research Partners’ or CHARPS [24]. The 883 trained volunteer CHARPS disseminated cancer awareness messages, resulting in statewide increases in breast and cervical cancer screening utilization in Mississippi and Alabama.

In church-based randomized trials, we evaluated three interventions aimed at increasing breast and colorectal cancer screening and informed decision-making for prostate cancer screening. ‘As You Go, Spread the Word,’ the breast cancer intervention, consisted of a brief educational booklet developed for church-attending African American women [25]. It resulted in significant increases in knowledge about mammograms, breast cancer, and treatment, as well as decreases in perceived barriers to mammography screening at the one-month follow-up assessment [25]. The ‘Brother to Brother Guide About Prostate Cancer and Screening’ consisted of a brief educational booklet and workshop led by CHAs in church settings [26]. This intervention resulted in significant changes from baseline to immediate follow-up in measures of prostate cancer knowledge and self-efficacy for informed decision-making about screening [26]. Finally, the ‘Take Charge of Your Health’ intervention was a two-part educational workshop series on colorectal cancer conducted in African American churches, with supplementary print materials [27]. The intervention resulted in significant increases in awareness of all four screening modalities, and in self-reported receipt of fecal occult blood test, flexible sigmoidoscopy, and colonoscopy [27]. With each of these interventions showing evidence of efficacy for their outcomes, the next step in the research program was to examine ways to extend the reach to include more churches and congregants.

### Role of technology in dissemination/implementation

Since the ‘dot-com boom’ of the 1990s, there has been a growing focus on eHealth – use of interactive technologies (*e.g*., the Internet, social media platforms, personal digital assistants, cellular phones, computer kiosks) – as platforms for health information dissemination, health-related behavior change, and decision-making [28-30]. The ‘Health Online 2013’ report by the Pew Foundation found that of the 81% of U.S. adults who use the Internet, 59% report using it to obtain health information [31]. Additionally, though research on eHealth initiatives is still growing, effective interventions have been documented over a wide array of health topics including, but not limited to, smoking cessation, weight management, anxiety and depression, and asthma management [29]. The Centers for Disease Control and Prevention’s web-based e-learning course for promoting engagement of community health workers is one such example of eHealth applications aiming to increase dissemination of an evidence-based curriculum [32]. Utilizing novel health communication efforts in today’s fast-changing technological environment increases the potential and capacity to close the gap between research discovery and program delivery [33,34]. In Project HEAL, we use technology in an effort to expand reach of the intervention from its original format.

### The present study

**Figure 1:**
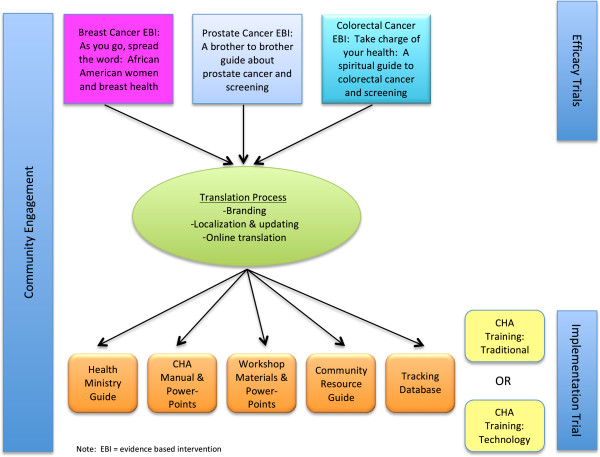
Project HEAL intervention translation process and components.

Project HEAL (Health through Early Awareness and Learning) is an implementation research project that aims to compare two strategies for implementing evidence-based cancer communication interventions in African American churches. We were unable to find previous studies that compared methods of training CHAs. The efficacy of in-person training for CHAs has previously been established [25-27]. Due to the need to close the gap between research and practice [7], the promising role that peer educators can play in dissemination [9], and the potential for technology to increase the reach of evidence-based interventions [29,32], we aimed to determine feasibility of a technology-based approach to training CHAs. A subsequent phase will assess whether the technology-based approach is comparable to the traditional classroom training for implementation outcomes in the delivery of an evidence-based curriculum. In the ‘traditional’ approach, peer CHAs are trained by study staff to implement a series of three educational workshops that cover breast, prostate and colorectal cancer screening. The CHAs receive as much technical assistance/support from study staff as they need. The second strategy, the ‘technology’ approach, is the same as the traditional except that the CHAs complete their training and certification independently using a web portal, and they receive minimal technical assistance/support from study staff (see Figure 1). We are unaware of previous technology-based methods to train lay CHAs. An aforementioned program [32] was not targeted to lay individuals but rather those with an existing health background, and was subject to a process evaluation including satisfaction. This article describes a community-engaged process of translating our series of three evidence-based cancer communication interventions into one coherent, branded strategy for training peer CHAs with these two delivery mechanisms. We start by describing the process, then outline the intervention components, report on the pilot test, and conclude with lessons learned from each of these phases.

## Methods

### Evidence-based interventions

In our three previous studies focusing on breast, prostate and colorectal cancer early detection among African Americans, we conducted separate cluster randomized trials with the church as the unit of analysis. This resulted in a set of three evidence-based cancer communication interventions with several strengths. The interventions are: (a) culturally appropriate and were designed with extensive community engagement and pilot testing; (b) theory-based, using the Health Belief Model [35] in both the intervention content and in the evaluation; (c) evidence-based, having been tested in group randomized trials for their impact on relevant cancer communication and behavioral outcomes; (d) spiritually-based, incorporating religious/spiritual content and themes; (e) designed for dissemination and external validity, meaning they require minimal translation to put into ‘real world’ practice; and (f) apply a peer CHA approach, which naturally builds organizational capacity and potentially facilitates sustainability.

### Translation process

All study methods were approved by the University of Maryland Institutional Review Board (#10-0691). Each previous intervention consisted of educational print materials developed specifically for the intended audience, a CHA training manual, CHA training materials, a PowerPoint presentation for CHA delivery in the church setting, and protocols for program administration and evaluation (see Figure 1). As such, the interventions were ‘manualized’ and developed with dissemination in mind. However, they needed to be combined into one package (*i.e*., one central training manual and protocol) through a translational process for Project HEAL. An exception is that the breast cancer project was not originally delivered using CHAs, and thus the breast cancer content module had to be developed. The intervention structures were otherwise uniform across projects and were combined into one coherent cancer early detection program, along with project branding. As shown in Table 1, workshop 1 is largely an orientation and enrollment session with introductory core cancer content, workshop 2 is a split session where men attend a prostate cancer session and women attend a breast cancer session, and workshop 3 is a combined workshop on colorectal cancer.

**Table 1:** Structure of Project HEAL intervention

**CHA training**	**CHA certification**	**↓ Church members/participants ↓**				
**↑ CHAs ↑**		Workshop 1: orientation/enrollment, cancer overview	Workshop 2: Men – Prostate; Women – Breast	Workshop 3: all – Colorectal	Individual-level maintenance	
Assessments (‘0’) ⇒		0		0	0	0
		Month 1	Month 2	Month 3	Month 12	Month 24
**⇐ Process Evaluation/Treatment Fidelity ⇒**						

The project team worked with an Advisory Panel of nine community stakeholders (*e.g*., faith leaders, healthcare system leaders, cancer survivors) to ready the interventions for implementation. These individuals were separate from the community partners. This helped to ensure that decisions made were relevant for the local context. The training manuals were merged into one, and the breast cancer CHA training module was developed based on procedures used to create the prostate and colorectal cancer modules in the earlier studies. Intervention protocols were merged into one, with a unified project name and logo. The Advisory Panel met on an as-needed basis and communicated electronically to provide advisement. They each received $200 for their time and participation.

**Figure 2:**
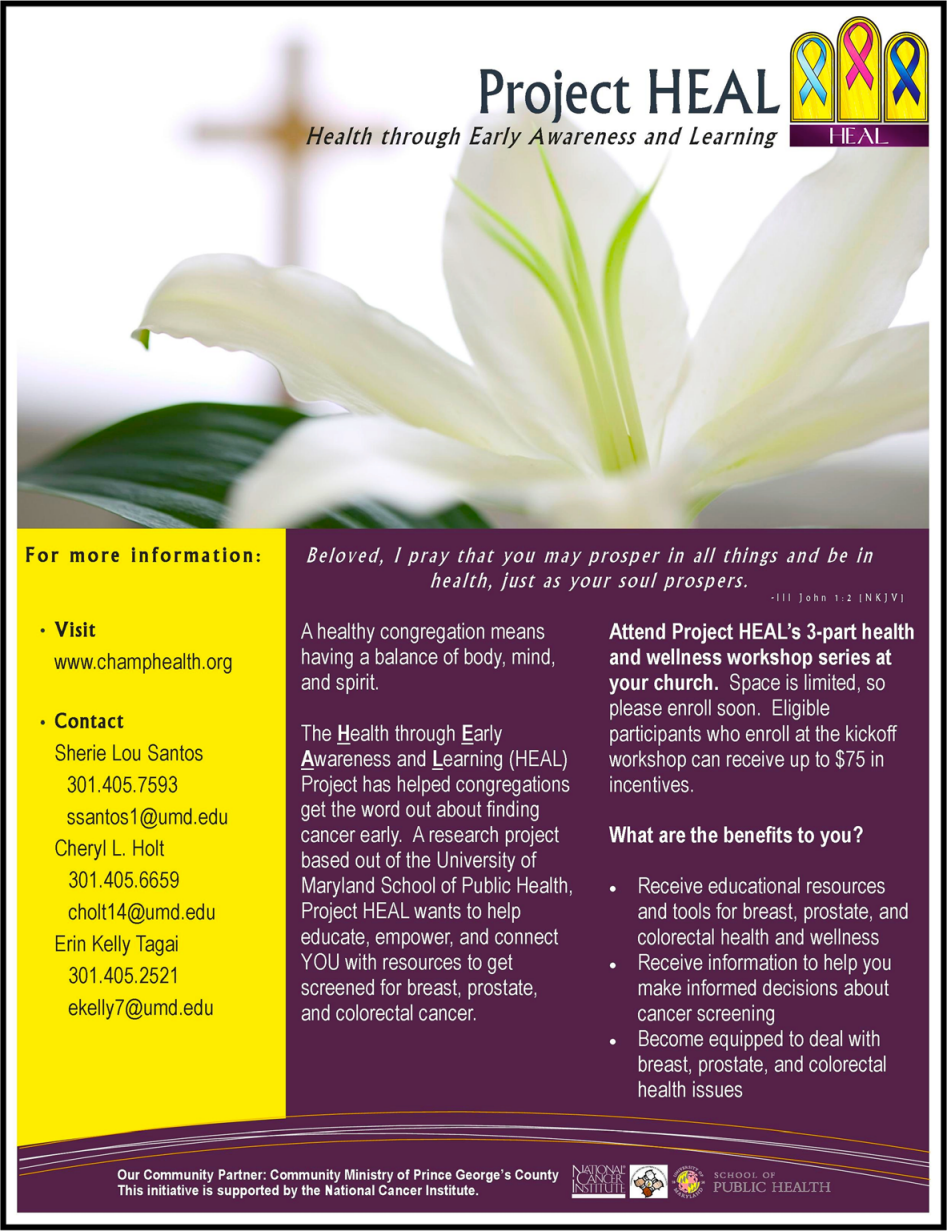
Participant recruitment flyer.

**Figure 3:**
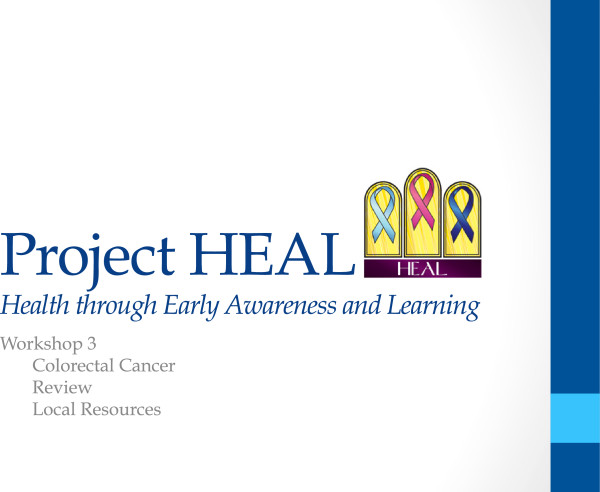
Workshop PowerPoint slide sample.

**Figure 4:**
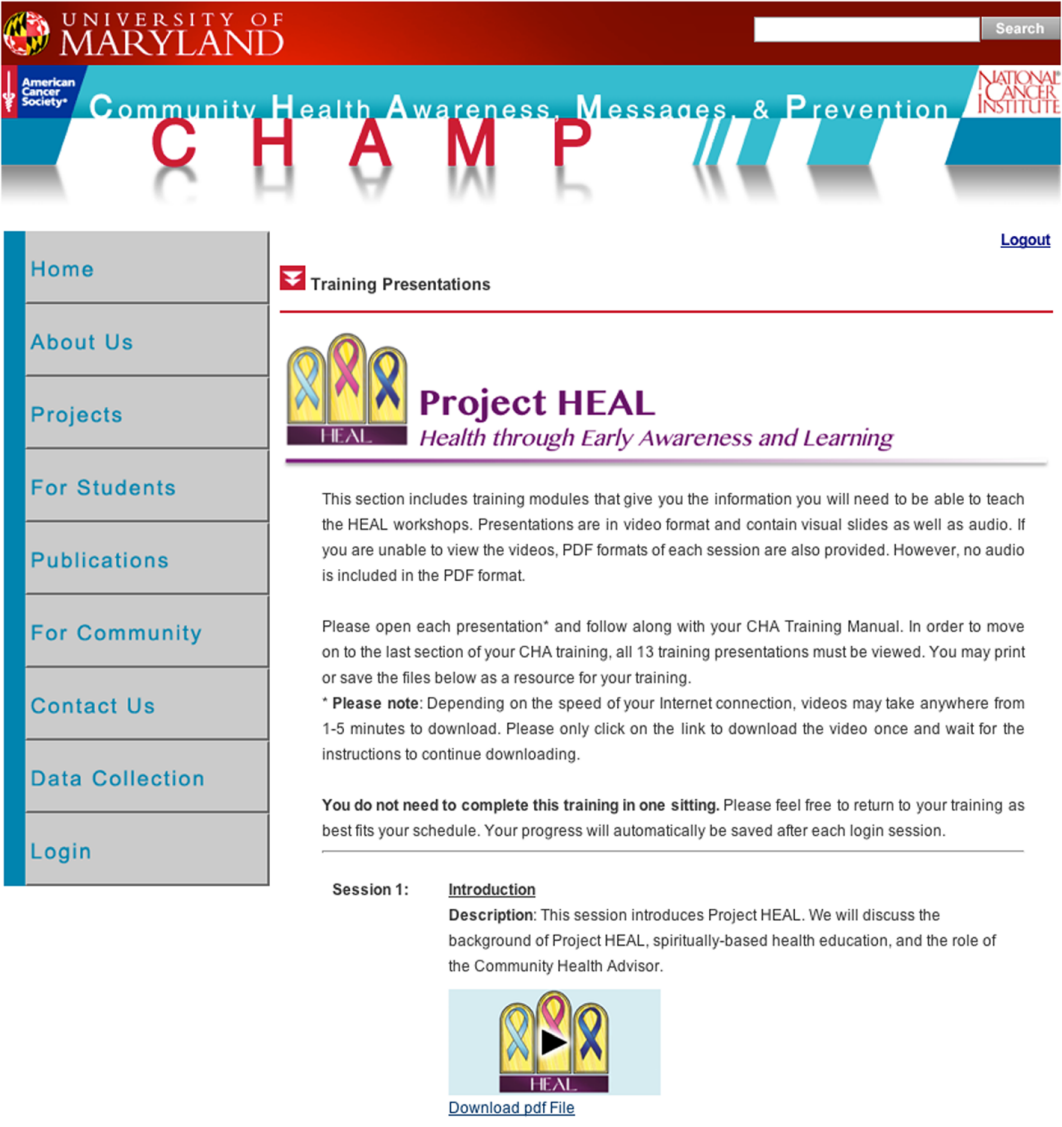
Web-based training portal sample.

The project team and Advisory Panel engaged in a process of project branding and identity formation (see Figures 2, 3 and 4). This included development of a community-friendly project name, logo, and promotional materials for use in project activities and recruitment (*e.g*., logo shirts, magnets). The logo and color scheme was applied to all project materials (*e.g*., recruitment fliers, training materials, surveys). The project became known as ‘Project HEAL’. The process concluded with all intervention materials and protocols readied for the piloting phase, where they were implemented in two churches, one in each study condition: traditional and technology (see section on piloting).

### Intervention components

#### *Health Ministry Guide*

The Health Ministry Guide was added for Project HEAL and is designed as a brief overview to introduce a faith-based organization to the project. It was modeled after that used in Body and Soul [36]. The Health Ministry Guide includes a welcome letter from the Principal Investigator, an overview of Project HEAL, benefits of participation, and a brief summary of early detection of breast, prostate and colorectal cancer. Information is also provided on planning for project success, how Project HEAL can be customized for each congregation, a recommended timeline, and how to plan for sustainability. Hard copies were distributed to church pastors as well as CHAs in both study conditions.

#### *CHA manual and PowerPoint modules*

The CHA manual and corresponding PowerPoint modules were used to train CHAs in the Project HEAL curriculum. It contains 13 modules (see Table 2).

**Table 2:** Project HEAL training modules

**Module**	**Description**
1. Introduction	Introduces Project HEAL and discusses the study background, spiritually-based health education, and the role of the Community Health Advisor (CHA).
2. Overview of cancer	Provides an introductory overview to what cancer is, how it develops, and the causes of cancer, including risk factors.
3. Breast cancer	Provides information about breast cancer, risk factors and symptoms, screening tests, and treatment options.
4. Prostate cancer	Provides information about prostate cancer, risk factors and symptoms, screening tests, informed decision-making, and treatment options.
5. Colorectal cancer	Provides information about colorectal cancer, risk factors and symptoms, screening tests, and treatment options.
6. Health beliefs	Discusses what influences individuals’ health beliefs, why people do not get screened for cancer, and covers common facts versus myths for breast, prostate and colorectal cancer.
7. Spirituality and health	Covers information describing how to bridge the gap between the church and the healthcare community, church-based health education, how scripture can motivate change in church-attenders, and examples of health-related scripture, relevant bible themes, and testimonials.
8. Adult education	Provides information on how to teach adult learners, the characteristics and unique needs of adult learners, and the differences between facts and opinions.
9. Leadership skills	Discusses what leadership is and details the characteristics of a good leader.
10. Communication skills	Covers what communication is, the importance of communication, communication skills, guidelines for effective speaking, how to prepare to speak, preparing the setting, and how to communicate your message.
11. Conducting the workshop	Discusses how to prepare for a Project HEAL workshop, including how to open, run, and close your workshop, how to handle the unexpected, practical tips for conducting a workshop, next steps for CHAs after training is completed, and a workshop plan of action.
12. Documentation	Introduces documentation and its purpose, as well as examples of documentation.
13. Ethical issues	Provides information about ethical issues in research, including the purpose of ethics in research, basic ethical principles, how people who participate in research are protected, and confidentiality and why it is important.

#### *Traditional CHA training*

CHAs trained in the traditional approach receive a spiral-bound hard copy of the manual and complete six hours of in-person training with approximately two additional hours of assigned self-study modules (‘leadership skills,’ ‘communication skills,’ ‘documentation,’ and ‘ethical issues’).

#### *Traditional CHA certification*

Following the roughly eight hours of training, CHAs in both groups complete a knowledge examination to determine their mastery of the curriculum. The multiple choice exam covers core content on breast, prostate and colorectal cancer (*e.g*., symptoms, risk factors, screening methods) and is delivered in paper-and-pencil format. CHAs must pass the examination with a score of 85% or better before becoming certified to conduct the Project HEAL workshop series. Should they not pass, they may take the examination again until they pass.

#### *Web-based CHA training portal*

After CHAs in the technology group are identified and recruited, they are provided with a user name and password by study staff. Additionally, they receive print copies of the spiral-bound CHA Training Manual, Health Ministry Guide, and Cancer Resource Guide to complement their online training process. They log into the system, read the informed consent and Memorandum of Understanding (replicas of the print versions used in the traditional approach), and indicate their agreement through an electronic signature system. Only then can they proceed to the training material. The CHA can then download the modules as a video developed from a narrated PowerPoint. The PowerPoint slides used in the online training portal and in the traditional approach are exact replicas with the exception of the narrations added for the web portal. This allows the CHA to hear audio and read the content, or use a PDF (portable document format) with written content only. They can log on and off the system as many times as needed until they reach completion. CHAs can use a computer and mobile devices equipped with iOS (*e.g*., iPad, iPhone) to engage with the training portal. When they have downloaded all of the training modules and a CHA training evaluation survey, only then can they enter the knowledge examination.

#### *Web-based CHA certification*

The online examination content and questions are exact replicas of the paper-based version administered to traditional CHAs. When they pass the examination with a score of 85% or better, they can download their certificate of completion and access all materials needed to conduct their workshops (*e.g*., workshop PowerPoint slides, sign-in sheet). Should they need technical assistance/support, contact information is provided for study staff.

#### *Workshop materials and PowerPoint slides*

The trained and certified Project HEAL CHAs are provided with standard PowerPoint slides to guide their presentations for each of their three workshops (cancer overview, breast/prostate cancer, and colorectal cancer). Workshops are delivered by the CHAs in their own church to eligible congregation members in an in-person educational session. Each workshop is approximately 1.5 hours in length and delivered approximately one month after CHA certification with each workshop scheduled in one-month intervals. Individuals are eligible to participate in the workshops if they: (a) self-identify as African American, (b) are between the ages of 40 and 75, (c) have no personal history of breast, prostate or colorectal cancer, (d) can attend all remaining workshops, and (e) are able to complete project surveys. In addition, the CHAs distribute a brief educational booklet on breast, prostate and colorectal cancer at the workshop in which this content is presented.

#### *Community resource guide*

We developed a local community cancer screening resource guide that lists the major providers in the study region, in accord with insurance coverage, or lack thereof. Information on treatment referral and cancer support groups was also identified. CHAs receive this information in training so that they are able to guide participants to appropriate local screening and treatment resources. The technology CHAs receive these materials with the hard copy of their manual prior to training as well as a PDF copy on the web training portal. All project manuals and protocols may be requested directly from the authors.

#### *Project management tracking database*

A tracking database was developed to allow for real-time information sharing between both university and community partners (community-based Project HEAL staff). This password protected database was developed on a Google Drive platform and is accessed through a central website. Organized by church, it provides status information organized by project phase (*e.g*., CHA training, workshop implementation). Using this database not only provides measures for process evaluation outcomes, but also allows for team information sharing in a secure and central location.

### Intervention pilot testing

#### *Web portal usability testing*

Usability testing was conducted to determine whether the web portal was easy for our pilot CHAs to use and navigate. Testing was completed with five pilot CHAs (three female and two male). The pilot CHAs were recruited from an African American church from the study setting (mid-Atlantic region). The pilot CHAs engaged with the training with a study staff member and were instructed to provide feedback or voice any issues as they went through the website. The testing took approximately 45 minutes to complete, and each participant received a $25 gift card at the end of the session. The pilot CHAs noted some confusion over the process of downloading the informed consent form, frustration over the slow download speed of the training videos, and indicated the location of the log in on the website was difficult to find. Modifications were made to the online training to address these issues and reduce confusion.

#### *Workshop pilot*

After all materials were finalized, we conducted a pilot test of the workshop implementation protocols and all CHA training and intervention materials. Similar to the Centers for Disease Control and Prevention’s process evaluation during pilot testing of their e-learning course [32], this brief period of piloting provided an opportunity for fine-tuning the protocols and CHA training procedures. We piloted each study condition (traditional and technology) with two CHAs each in two separate churches. Both churches’ CHAs received the entire CHA training curriculum. However, in accord with the study timeline, the traditional condition church was only asked to deliver the breast/prostate cancer workshop, and the technology condition church was asked to deliver the colorectal cancer workshop.

### 1. Pilot CHA training – traditional approach

The traditional CHA training was conducted at the CHAs’ church over two three-hour sessions, with training modules presented as PowerPoint slides by six members of the Project HEAL team and an Advisory Panel member. At the conclusion of the pilot, revisions were made to the PowerPoint slides to reduce redundancy and maximize efficiency.

### 2. Pilot CHA training – technology approach

After study staff received the names of the CHAs from the Pastor, the technology CHAs were sent instructions and access to the web portal by email. The CHAs were to complete the training with minimal assistance from study staff. It became apparent over several weeks that while the CHAs expressed interest, one pilot CHA had not logged into the system to start the training, and the other CHA had slow progress. After study staff and church officials had difficulty contacting the CHA that had not yet logged into the system, a decision was made to identify a new CHA. The study team concluded that an in-person orientation and technical assistance meeting with the CHAs may be necessary to orient them to the online system, build rapport, and initiate the training. A meeting approximately one hour in length was scheduled with the CHAs and church officials. The web portal was introduced to the new CHA, and logistics about the training and scheduling their workshop were discussed. After the meeting, both CHAs successfully completed their training within approximately one month.

### 3. Pilot workshop – traditional approach

After the CHAs were certified, they began the intervention protocol and were asked to conduct the breast/prostate cancer workshop. Workshops began with informed consent and a baseline participant survey. Ten eligible participants attended the workshop (60% male). The mean age of the participants was 69.3 (SD = 11.81). These participants completed the baseline survey and a one-time follow-up on-site assessment and received a $25.00 store gift card for doing so. In accord with the workshop series protocol, the CHAs began the workshop together and separated into breakout sessions where the male CHA led the prostate cancer session and the female CHA led the breast cancer session.

Several observations were noted from the pilot workshop. Though the first workshop in the three-part series was dedicated to enrollment and kickoff, the completion of informed consent and the baseline survey took more time than anticipated, relative to the educational component. Therefore, the recommendation was to streamline the survey and add cancer content slides to the PowerPoint presentation. To avoid participants leaving the workshop before completion of the post-workshop survey and receiving their gift card, a note was made to ask participants to sign in and sign out, to ensure that they received the incentive.

### 4. Pilot workshop – technology approach

These CHAs were asked to conduct the colorectal cancer workshop. A total of 18 eligible participants attended the workshop (78% female). The mean age of the participants was 61.6 (SD = 8.56). Following the protocol design for this session, both CHAs co-presented the information to a combined audience of both male and female participants.

CHAs in both study groups followed the protocol for the workshops. Observations from this pilot workshop included the need for increased font size on the PowerPoint sides and additional detail on the individual screening methods for colorectal cancer. It was clear that while the CHAs gave an outstanding and engaging presentation, they were not as well suited for tasks like assisting study staff with screening participants for eligibility. It was confirmed that study staff would handle all enrollment and screening procedures. Finally, both workshops drew interest from cancer survivors, who were not eligible to participate given the focus on screening. To offer support and encouragement, the team decided to add a survivor component to the workshop agenda, giving the opportunity to receive recognition and to briefly share their testimonies.

### Implementation trial

In the following trial phase, 14 churches will be recruited and then randomly assigned to receive one of these two strategies in a 1:1 allocation ratio, and will be followed over a two-year period to assess sustainability. As is shown in Table 1, the workshops are planned for a 6-month implementation phase, with follow-ups at 12 and 24 months.

Overall, Project HEAL is designed to answer several questions relevant to implementation research. First, we wanted to determine whether our series of three evidence-based cancer communication interventions could be successfully translated (focus of the current report), and then implemented and sustained in African American church settings. Second, we wanted to examine two approaches to CHA training, traditional vs. technology, to determine which level of technical assistance would be optimal for successful implementation and sustainability. We hypothesized that those churches with strong organizational capacity [37] would have successful implementation and sustainability outcomes under both CHA training approaches. However, those with less organizational capacity were expected to do well only under the traditional training approach, due to the critical role of technical assistance in such environments. We wanted to determine whether the technology-based training would be feasible and effective for the study implementation outcomes based on the RE-AIM Framework [38]. Due to lack of guidance from previous research, we felt it was premature to propose specific hypotheses on differences between methods of CHA trainings in the implementation outcomes.

The RE-AIM Framework [38] will be used in Project HEAL evaluation. Reach, or the extent to which participants are involved, will be assessed by the percent of eligible congregation members that enroll in the project and attend workshops. Efficacy, or the success rate of the intervention, will be assessed through Health Belief Model [35] outcomes and self-reported screening, measured at post-workshop assessments and also at 12- and 24-month follow-ups. Adoption, or the proportion of settings that adopted Project HEAL, will be assessed as the church cooperation rate (percent) of all the churches contacted during the church recruitment phase. Implementation is assessed through a number of process outcomes including CHA training, adherence to workshop protocol (assessed through a workshop observation checklist by research staff for each workshop), and self-report of program modifications. Maintenance, or the extent to which Project HEAL is sustained over time, will be assessed through CHA interviews after the workshops and at 12 and 24 months. Implementation and maintenance/sustainability data will be gathered from multiple perspectives, such as the CHAs, participants, pastors, project team observations, and the project tracking system. Finally, to assess organizational capacity, we will conduct Pastor interviews in each church in order to collect detailed information on each congregation (*e.g*., size, ministries, staffing). The CHA training itself is evaluated as to whether the CHAs completed it, whether they conducted the workshops, and whether those workshops resulted in outcomes (*e.g*., increased knowledge, screening) among attendees.

## Discussion

Project HEAL is designed to address implementation research questions in community-based settings and within a health disparities context. In that way, the project is poised to make a contribution to the dissemination/implementation literature, while the use of technology could have implications for broader reach. This article describes the community-engaged process of translating from our series of three original evidence-based cancer communication interventions into one coherent, branded approach, with two delivery mechanisms. The application of two training conditions is somewhat unique as there are a limited number of published studies that incorporate multiple approaches to CHA training [39]. This process resulted in a number of lessons learned and modifications to the original planned approach. Two main lessons stand out with regard to the intervention itself. First, the overall process took longer than originally anticipated. There were several reasons for this. The materials had to be combined from three different interventions and blended into one seamless package for Project HEAL. As the three previous interventions had materials in different formats, time had to be spent in reformatting. Additionally, as Project HEAL was implemented in a different cultural and geographic setting, the materials had to be assessed for the new setting (*e.g*., scripture in either New King James Version or New International Version). New materials development such as the breast cancer training module, Health Ministry Guide, and online training portal also added to the overall timeline. Lastly, the cancer statistics, screening guidelines, and medical information had to be updated.

Second, in the technology condition, the team had concerns about the delays in completion of the pilot CHA training. For this particular church, it was necessary to spend some time building a relationship with the technology group CHAs prior to their engagement with the training portal. It may have been unrealistic to expect them to respond to an email from a stranger to log onto an online system and complete several hours of training even if they were asked to do so by their church leadership. The individual orientation and technical assistance meeting with the CHAs not only serves to get them familiarized with the web portal, but also gives them a human point of contact with the project. Moving forward into our implementation trial, a two-week time period will be introduced into the protocol to allow CHAs time to engage in the training process independently. After two weeks, the project team will offer a technical assistance orientation to the CHAs if needed. This in-person contact may have implications for the technology approach in terms of future reach and translation capabilities. A cancer screening program using technology and promotoras noted similar experiences with technology uptake [39]. While the technology was not incorporated into the promotoras’ training, it was utilized for program delivery. The promotoras experienced challenges with the more advanced technology that required password memorization, new skills, and these difficulties may be associated with limited educational or socioeconomic status.

The piloting also resulted in modifications regarding data collection protocols. The team expressed concerns about survey length, which were confirmed in the pilot. Participants spent significant time on the survey and gave the questions serious consideration. It was clear that the survey battery had to be made shorter.

### Roles and expectations

It was initially anticipated that the CHAs might be able to assist the study team with tasks such as participant pre-enrollment and eligibility screening. However, it became apparent in the pilot that these tasks were better suited for the study team. In a process evaluation of the Body and Soul intervention to increase fruit and vegetable and decrease fat consumption in African American churches [36], it was reported that survey processes placed a burden on church liaisons [40]. The CHAs also have an important role with regard to capacity building and sustainability. For example, though the two pilot churches were not required to conduct the other workshops in the series as part of the pilot, they both chose to do so on their own. In the Body and Soul process evaluation, project staff expressed that without staff and resources, it would be unlikely that the program would be sustained in the churches [40]. They recommended policy changes such as setting up permanent church committees to increase long-term maintenance. Churches were reported to have a more difficult time implementing complex tasks that required resources and skills that may not be readily available or frequently used in their church [41,42]. It was suggested that future work might require additional protocols for technical assistance [41] and designated staffing to assist churches with complex tasks [42].

### Limitations

The current study may be limited by a number of factors. As with any community-based study, the situations we encountered may not be generalizable beyond the present context. The lessons learned from the churches involved in the pilot study reflect a new foray into the technology-based method of CHA training and need further verification. The Project HEAL trial will be informative in terms of feasibility of this method of training for the CHAs. Also relating to the technology training approach, unlike the traditional approach, it is not known how the CHAs will interface with the material other than that they have to download it and pass a knowledge examination to become certified. Furthermore, there was no opportunity to observe CHAs’ performance prior to their conducting the workshops. While the technology approach may provide greater reach, lower cost, and more flexibility, it is possible that it may not be as intensive or even effective for producing qualified and confident CHAs to initiate and schedule the workshops. This again is something that will be tested in the upcoming trial. It is possible that the technology approach may provide certain advantages, but we may learn that technical assistance such as a pre-training face-to-face meeting, having previously trained CHAs provide peer support, or provision of ‘frequently asked questions’ information may be needed.

## Conclusions

Project HEAL is a promising implementation research study with several contributions. We report on intervention translation and development of a technology component to deliver CHA training to lay individuals in faith-based organizations. If found to be feasible in the larger trial, there may be wider implications geographically and for broader chronic disease prevention and control. Finding ways to promote evidence-based interventions in faith or other community-based organizations is highly significant. Using community-engaged research processes to discover how to do this more effectively can make a positive and sustainable impact on health disparities.

## Abbreviations

HEAL: Health through Early Awareness and Learning; CHA: Community health advisor; PDF: Portable document format; SD: Standard deviation; RE-AIM: Reach effectiveness adoption implementation maintenance; EBI: Evidence-based intervention.

## Competing interests

The authors declare that they have no competing interests.

## Authors’ contributions

CLH conceived and provided scientific and administrative leadership and coordination of the study; drafted much of the manuscript; and approved all edits. EKT drafted sections of the manuscript, played a lead role in developing the online CHA training, and assisted with data collection and management. MAS made substantial contributions to the design of the study as it relates to organizational theory, implementation and sustainability, and provided critical reviews of the manuscript. SLZS played a leadership role in the overall implementation and management of the project, and played a lead role with ET in developing the online CHA training. MQW contributed to the study design and evaluation plan, designed and managed the web-based training module, and conducted statistical analyses. JB played a role in intervention development activities, including participant recruitment and retention protocols, and provided critical reviews of the manuscript. MH and TW contributed to study’s community engagement and evaluation plan with a particular focus on process evaluation and fidelity. JLS served as a community researcher and played a critical role in providing guidance in study decision-making in issues relating to the faith community (*e.g*., recruitment, feasibility, data collection protocols). All authors were involved in the interpretation and discussion of results; contributed to the writing and review of the various drafts of the manuscript; and read and approved the final manuscript.

## References

[B1] World Health Organization Cancer fact sheet http://www.who.int/mediacentre/factsheets/fs297/en/

[B2] Campbell MK Hudson MA Resnicow K Blakeney N Paxton A Baskin M Church-based health promotion interventions: evidence and lessons learned Annu Rev Publ Health 2007 28 213 234 10.1146/annurev.publhealth.28.021406.144016 17155879

[B3] Husaini BA Reece MC Emerson JS Scales S Hull PC Levine RS A church-based program on prostate cancer screening for African American men: reducing health disparities Ethnic Dis 2008 18 2 S2 S179 184 18646345

[B4] Zahuranec DB Morgenstern LB Garcia NM Conley KM Lisabeth LD Rank GS Smith MA Meurer WJ Resnicow K Brown DL Stroke health and risk education (SHARE) pilot project: feasibility and need for church-based stroke health promotion in a bi-ethnic community Stroke 2008 39 1583 1585 10.1161/STROKEAHA.107.503557 18323486

[B5] Sauaia A Sung-joon M Byers T Lack D Apodaca C Osuna D Stowe A McGinnis GF Latts L Church-based breast cancer screening education: impact of two approaches on Latinas enrolled in public and private health insurance plans Prev Chronic Dis 2007 4 A99 17875274 PMC2099296

[B6] Darnell JS Chang CH Calhoun EA Knowledge about breast cancer and participation in a faith-based breast cancer program and other predictors of mammography screening among African American women and Latinas Health Promot Pract 2006 7 Suppl 3 201S 212S 16760248 10.1177/1524839906288693

[B7] Glasgow RE Marcus AC Bull SS Wilson KM Disseminating effective cancer screening interventions Cancer 2004 101 Suppl 5 1239 1250 15316911 10.1002/cncr.20509

[B8] Kerner JF Integrating research, practice, and policy: what we see depends on where we stand J Public Health Manag and Pract 2008 14 193 198 10.1097/01.PHH.0000311899.11197.db 18287927

[B9] Diffusion and dissemination of evidence-based cancer control interventions http://www.ahrq.gov/clinic/epcsums/canconsum.htm PMC4781142 12794961

[B10] Weiner BJ Lewis MA Linnan LA Using organization theory to understand the determinants of effective implementation of worksite health promotion programs Health Educ Res 2009 24 292 305 18469319 10.1093/her/cyn019

[B11] Ammerman A Process evaluation of the church-based PRAISE! Project: partnership to reach African Americans to increase smart eating 2002 1 San Francisco: John Wiley & Sons, Inc

[B12] Kerner JF Guirguis-Blake J Hennessey KD Brounstein PJ Vinson C Schwartz RH Myers BA Briss P Translating research into improved outcomes in comprehensive cancer control Cancer Cause Control 2005 16 Suppl 1 27 40 10.1007/s10552-005-0488-y 16208572

[B13] Witmer A Seifer SD Finocchio L Leslie J O’Neil EH Community health workers: integral members of the health care work force Am J Public Health 1995 85 1055 1058 10.2105/AJPH.85.8_Pt_1.1055 7625495 PMC1615805

[B14] U.S. Department of Health and Human Services Community health advisors: models, research, and practice, selected annotations-United States. Volume 1 1994 Atlanta; U.S: Department of Health and Human Services

[B15] U.S. Department of Health and Human Services Community health advisors: programs in the United States, health promotion and disease prevention 1994 Atlanta; U.S: Department of Health and Human Services

[B16] Fendall R We expect too much from community health workers World Health Forum 1984 5 300 303

[B17] Walt G Community health workers in national programmes. Just another pair of hands? 1990 Philadelphia: Open University Press

[B18] Indian Health Service Alaska community health aide program description 1991 Washington: Government Printing Office

[B19] Giblin PT Effective utilization and evaluation of indigenous health care workers Public Health Rep 1989 104 361 368 2502807 PMC1579943

[B20] Richter RW Bengen B Alsup PA Bruun B Kilcoyne MM Challenor BD The community health worker: a resource for improved health care delivery Am J Public Health 1974 64 1056 1061 10.2105/AJPH.64.11.1056 4416129 PMC1775649

[B21] Yu MY Song L Seetoo A Cai C Smith G Oakley D Culturally competent training program: a key to training lay health advisors for promoting breast cancer screening Health Educ Behav 2007 2007 34 928 941 17965228 10.1177/1090198107304577

[B22] Maxwell AE Danao LL Cayetano RT Crespi CM Bastani R Evaluating the training of Filipino American community health advisors to disseminate colorectal cancer screening J Comm Health 2012 37 1218 1225 10.1007/s10900-012-9557-9 PMC3383892 22430865

[B23] Navarro AM Raman R McNicholas LJ Loza O Diffusion of cancer education information through a Latino community health advisor program Prev Med 2007 45 135 138 10.1016/j.ypmed.2007.05.017 17604831 PMC2043119

[B24] Lisovicz N Johnson RE Higginbotham J Downey JA Hardy CM Fouad MN Hinton AW Partridge EE The deep South network for cancer control: building a community infrastructure to reduce cancer health disparities Cancer 2006 107 Suppl 8 1971 1979 16921494 10.1002/cncr.22151

[B25] Holt CL Klem PR As you go, spread the word: spiritually based breast cancer education for African American women Gynecol Oncol 2005 99 Suppl 1 S141 S142 16139347 10.1016/j.ygyno.2005.07.066

[B26] Holt CL Wynn TA Litaker MS Southward P Jeames S Schulz E A comparison of a spiritually-based and a non-spiritually based educational intervention for informed decision making for prostate cancer screening among church-attending African American men Urol Nurs 2009 29 249 258 19718941 PMC2836722

[B27] Holt CL Litaker MS Scarinci IC Debnam KJ McDavid C McNeal SF Eloubeidi MA Crowther M Bolland J Martin MY Spiritually-based intervention to increase colorectal cancer screening among African Americans: screening and theory-based outcomes from a randomized trial Health Educ Behav 2013 40 458 468 10.1177/1090198112459651 23033548 PMC5568036

[B28] Eng TR eHealth research and evaluation: challenges and opportunities J Health Commun 2002 7 267 272 10.1080/10810730290001747 12356287

[B29] Strecher V Internet methods for delivering behavioral and health-related interventions (eHealth) Annu Rev Clin Psychol 2007 3 53 76 10.1146/annurev.clinpsy.3.022806.091428 17716048

[B30] Turner-Lee N Smedley B Miller J Minorities, Mobile broadband and the management of chronic diseases http://www.jointcenter.org/sites/default/files/upload/research/files/Minorities%20Mobile%20Broadband%20and%20the%20Management%20of%20Chronic%20Diseases_0.pdf

[B31] Fox S Duggan M Health online 2013 http://www.pewinternet.org/~/media//Files/Reports/PIP_HealthOnline.pdf

[B32] Brownstein JN Mirambeau AM Roland KB News from the CDC: using web-based training to translate evidence on the value of community health workers into public action Transl Behav Med 2013 3 229 230 10.1007/s13142-013-0204-5 24073171 PMC3771017

[B33] Chou WY Prestin A Lyons C Wen KY Web 2.0 for health promotion: reviewing the current evidence Am J Public Health 2013 103 e9 e18 23153164 10.2105/AJPH.2012.301071 PMC3518341

[B34] Griffiths F Lindenmeyer A Powell J Lowe P Thorogood M Why are health care interventions delivered over the internet? a systematic review of the published literature J Med Internet Res 2006 8 e10 10.2196/jmir.8.2.e10 16867965 PMC1550698

[B35] Rosenstock IM Strecher VJ Becker MH Social learning theory and the health belief model Health Educ Behav 1988 15 175 183 10.1177/109019818801500203 3378902

[B36] National Cancer Institute Body & Soul: A celebration of healthy eating & living, a guide for your church [http://rtips.cancer.gov/rtips/viewProduct.do?productId=257631&viewMode=product]

[B37] Greenhalgh T Robert G MacFarlane F Bate P Kyriakidou O Diffusion of innovations in service organizations: systematic review and recommendations Milbank Q 2004 82 581 629 10.1111/j.0887-378X.2004.00325.x 15595944 PMC2690184

[B38] Glasgow RE Vogt TM Boles SM Evaluating the public health impact of health promotion interventions: the RE-AIM framework Am J Public Health 1999 89 1322 1327 10.2105/AJPH.89.9.1322 10474547 PMC1508772

[B39] Arvey SR Fernandez ME LaRue DM Bartholomew LK When promotoras and technology meet: a qualitative analysis of promotoras’ use of small media to increase cancer screening among South Texas Latinos Health Educ Behav 2012 39 352 363 10.1177/1090198111418110 21986243 PMC3358563

[B40] Campbell MK Resnicow K Carr C Wang T Williams A Process evaluation of an effective church-based diet intervention: body & soul Health Educ Behav 2007 34 864 880 17200096 10.1177/1090198106292020

[B41] Allicock M Campbell MK Valle CG Carr C Resnicow K Gizlice Z Evaluating the dissemination of body & soul, an evidence-based fruit and vegetable intake intervention: challenges for dissemination and implementation research J Nutr Educ Behav 2012 44 530 538 10.1016/j.jneb.2011.09.002 22406012 PMC3374882

[B42] Baruth M Wilcox S Laken M Bopp M Saunders R Implementation of a faith-based physical activity intervention: insights from church health directors J Community Health 2008 33 304 312 10.1007/s10900-008-9098-4 18473154

